# Reconstruction of Hematopoietic Inductive Microenvironment after Transplantation of VCAM-1-Modified Human Umbilical Cord Blood Stromal Cells

**DOI:** 10.1371/journal.pone.0031741

**Published:** 2012-02-23

**Authors:** Yao Liu, Xing-hua Chen, Ying-jian Si, Zhong-jun Li, Lei Gao, Li Gao, Cheng Zhang, Xi Zhang

**Affiliations:** 1 Department of Hematology, Xinqiao Hospital, The Third Military Medical University, Chongqing, China; 2 Department of Blood Transfusion, Xinqiao Hospital, The Third Military Medical University, Chongqing, China; 3 Department of Pediatric Hematology/Oncology, BaYi Children's Hospital, The Military General Hospital of Beijing, Beijing, China; University of Sao Paulo – USP, Brazil

## Abstract

The hematopoietic inductive microenvironment (HIM) is where hematopoietic stem/progenitor cells grow and develop. Hematopoietic stromal cells were the key components of the HIM. In our previous study, we had successfully cultured and isolated human cord blood–derived stromal cells (HUCBSCs) and demonstrated that they could secret hemopoietic growth factors such as GM-CSF, TPO, and SCF. However, it is still controversial whether HUCBSCs can be used for reconstruction of HIM. In this study, we first established a co-culture system of HUCBSCs and cord blood CD34^+^ cells and then determined that using HUCBSCs as the adherent layer had significantly more newly formed colonies of each hematopoietic lineage than the control group, indicating that HUCBSCs had the ability to promote the proliferation of hematopoietic stem cells/progenitor cells. Furthermore, the number of colonies was significantly higher in vascular cell adhesion molecule-1 (VCAM-1)-modified HUCBSCs, suggesting that the ability of HUCBSCs in promoting the proliferation of hematopoietic stem cells/progenitor cells was further enhanced after having been modified with VCAM-1. Next, HUCBSCs were infused into a radiation-damaged animal model, in which the recovery of hematopoiesis was observed. The results demonstrate that the transplanted HUCBSCs were “homed in” to bone marrow and played roles in promoting the recovery of irradiation-induced hematopoietic damage and repairing HIM. Compared with the control group, the HUCBSC group had significantly superior effectiveness in terms of the recovery time for hemogram and myelogram, CFU-F, CFU-GM, BFU-E, and CFU-Meg. Such differences were even more significant in VCAM-1-modified HUCBSCs group. We suggest that HUCBSCs are able to restore the functions of HIM and promote the recovery of radiation-induced hematopoietic damage. VCAM-1 plays an important role in supporting the repair of HIM damage.

## Introduction

The hematopoietic inductive microenvironment (HIM) is where hematopoietic stem/progenitor cells (HSCs/HPCs) grow and develop [1]. Hematopoietic stromal cells, one of the key components of the HIM, primarily exist in tissues and organs such as bone marrow, spleen, and thymus [2]. Through direct contact with HSCs/HPCs and secretion of pluripotent hematopoietic growth factors (HGFs) and extracellular matrix (ECM), hematopoietic stromal cells not only are associated with the homing, proliferation, differentiation, and self-renewal of HSCs/HPCs but also play important roles in the occurrence, progression, and prognosis of some hematologic diseases [3], [4].

After stem cell transplantation, the ability of adherent bone marrow stromal cells of patients pre-treated with radiotherapy and chemotherapy have reduced ability to support the growth of HSCs/HPCs [5]. Some biological factors, such as cytomegalovirus, hepatitis B virus, and human immunodeficiency virus as well as physical and chemical factors, such as radiation and chemotherapeutic drugs, can cause hematopoietic dysfunction through the damage of stromal cells. In some disease states such as aplastic anemia, acute and chronic myeloid leukemia, and myelodysplastic syndrome, the abnormal hematopoietic function is associated with dysfunction of HSCs/HPCs as well as the number of stromal cells or dysfunction of stromal cells in the bone marrow HIM [5], [6], [7], [8], [9].

The hematopoietic dysfunction caused by damage of stromal cells in the HIM is longer-lasting than damage of parenchymal cells and, in fact, can be irreversible [10]. Therefore, repair or reconstruction of normal HIM function has become clinically challenging.

Autologous infusion of cultured and expanded bone marrow stromal cells is an effective ancillary method for repairing damaged hematopoietic function in experimental and clinical studies. The extensive use of hematopoietic stromal cells in the clinic is limited due to the dysfunction of the microenvironment in autologous bone marrow stromal cells in patients with hematopoietic disorders or due to immune-related problems such as graft-versus-host disease (GVHD) from allogeneic stromal cell implantation; moreover, the clinical values of fetal liver, thymus, and other tissue-derived stromal cells are hampered by ethical considerations. Therefore,searching for new sources of hematopoietic stromal cells that are convenient, healthy, and universally applicable is a topic of intense interest. HSCs/HPCs in umbilical cord blood are more primitive and have the advantages of a higher proliferation rate and more rapid hematopoietic reconstruction than those in bone marrow and peripheral blood. In addition, GVHD after cord blood transplantation usually is mild, and the graft-versus-leukemia effect will not be an issue; therefore, even HLA-incompatible cord blood can be successfully transplanted without ethical concerns [11]. Hematopoietic stromal cells mainly exist in bone marrow; however, it is still controversial whether cord blood contains stromal cells that can be used for reconstruction of HIM. Our team had cultured adherent cells from cord blood using Dexter's culture system. After identification by their surface markers, these cells have the biological characteristics of stromal cells and secreted HGFs; therefore, they were named human cord blood–derived stromal cells (HUCBSCs) [12]. In subsequent studies, our team further discovered that HUCBSCs not only promoted the reconstruction of erythroid and other hematopoietic lineages *ex vivo*, but interestingly, they also had a stronger ability to promote megakaryocyte proliferation than bone marrow stromal cells [13], [14]. In addition, they inhibited the occurrence of GVHD to some extent [15], [16]. The above results led us to believe that HUCBSCs might be a promising new source of hematopoietic stromal cells.

During the process of HSC transplantation, the homing of stem/progenitor cells is a major factor determining the success of transplantation. The molecular basis of the homing of HSCs/HPSs is specifically mediated by corresponding receptors and ligands of cell adhesion molecules (CAMs) expressed by hematopoietic parenchymal and stromal cells. The specific interaction between α4β1 integrin expressed by parenchymal cells and vascular cell adhesion molecule-1 (VCAM-1), an immunoglobulin (Ig) superfamily member expressed by bone marrow stromal cells, plays a key role in the adhesion of stem cells to the bone marrow stroma. Anti-α4β1 or anti-VCAM-1 monoclonal antibodies can block the adhesion of HSCs/HPCs to the adherent layer of bone marrow stromal cells [17]. The level of expression of VCAM-1 in hematopoietic stromal cells can affect the homing of HSCs, thus further affecting the process of hematopoietic reconstruction. Our previous studies showed that VCAM-1 expression in HUCBSCs was lower than that in bone marrow stromal cells, which might affect its ability to regulate hematopoiesis [18] To investigate the effect of the VCAM-1 gene on the ability of HUCBSCs in regulating hematopoiesis, a VCAM-1 high-expressing vector had been constructed using recombinant DNA technology and successfully transfected into HUCBSCs [19], which provided an effective tool and vector to study the function of VCAM-1–modified HUCBSCs in the regulation of hematopoiesis.

In this study, we first established a co-culture system of VCAM-1–modified HUCBSCs and cord blood CD34^+^ cells and then determined that VCAM-1–modified HUCBSCs had a stronger ability to support hematopoiesis *in vitro*. Next, VCAM-1–modified HUCBSCs were infused into a radiation-damaged animal model, in which the recovery of hematopoiesis was observed. The results demonstrate that transplantation of VCAM-1–modified HUCBSCs could repair functional damage to HIM and promote recovery from hematopoietic damage.

## Materials and Methods

### Source of cord blood

Twenty samples of cord blood were taken from umbilical cords of full-term normal-delivery babies delivered by healthy mothers at Xinqiao Hospital, Chongqing, China. Written informed consent was obtained from mothers in all cases and the ethics committee of Xinqiao Hospital approved the study. Heparin was used as the anti-coagulant. The cord blood volume collected ranged 60–100 ml.

### Source of animals and establishment of a hematopoietic damage model

Fifteen male and fifteen female BALB/c-nu/nu nude mice (aged 10–12 weeks, 20–25 g body weight) were purchased from the Third Military Medical University Laboratory Animal Center. Animals were housed in specific pathogen–free (SFP) rooms of the Laboratory Animal Center in the Second Affiliated Hospital of Third Military Medical University. Four ^60^Co-irradiation groups (3.5 Gy, 5 Gy, 6.5 Gy, and 8 Gy) were established to simulate mild, moderate, and severe damages of hematopoietic function to produce models for HIM damage. The animal experiments were also approved by the ethics committee of Xinqiao Hospital. The permit number is No. 20090205.

### Culture of HUCBSCs [12]

The mononuclear cells from human cord blood were obtained by Ficoll density gradient fractionation columns (density = 1.077 g/l Pharmacia Biotech, Uppsala, Sweden). CD34^+^ cells were separated using the magnetic cell sorting (MACS) system (Miltenyi Biotec; Bergisch Gladbach, Germany) according to the manufacturer's instructions, and percent purity of the positive fraction was determined using anti-CD34 fluorescein isothiocyanate (FITC) and phycoerythrin (PE) conjugate-tagged antibody (Santa Cruz, CA, USA) by flow cytometer (Becton Dickinson, Franklin Lakes, NJ, USA). CD34^+^ cells were resuspended in DMEM medium (Gibco, USA) containing 12.5% (v/v) fetal bovine serum (Hyclone, USA), 12.5% (v/v) horse serum (Gibco, USA), 10^−6^ M hydrocortisone, 10 ng/ml SCF (Sigma, USA), and 1 ng/ml bFGF (Sigma, USA). Culture was replaced by fresh medium after 48 h, and then, it was demi-depopulated weekly and fresh medium was added to it. After the density of cells had risen above 80% confluence, HUCBSCs were subcultured with 1∶1 ratio under the same culture medium and condition.

### Plasmid construction, transfection, and identification [19]

Using recombinant DNA technology, the target gene, VCAM-1, was cloned into a shuttle plasmid containing an EGFP reporter gene. Homologous recombination was performed between the recombinant plasmid and adenovirus genome in BJ5183 cells to produce recombinant adenoviruses. The recombinant adenovirus-expressing plasmid was then transfected into 293 cells mediated by liposomes to allow the packaging and replication of adenoviruses. The transfected 293 cells were observed under a fluorescence microscope, and the culture supernatant was used to detect the expression of target protein using western blot analysis. The VCAM-1 gene was then introduced into HUCBSCs using a highly efficient delivery system, and the expression of VCAM-1 was detected. Supernatant was then discarded, and cells were washed twice with culture medium.

### Observation of the effect of HUCBSCs on the in vitro proliferation of cord blood CD34^+^ cells

Human cord blood CD34^+^ cells were diluted in Iscove's Modified Dulbecco's Medium (IMDM) containing 12.5% (v/v) horse serum, 12.5% fetal calf serum, and 10^−6^ mol/L hydrocortisone at 2×10^5^ cells/ml in culture flasks containing a monolayer of HUCBSCs. The control group was cord blood CD34^+^ cells without expanded HUCBSCs. Cells were cultured at 37°C in a 5% CO_2_ incubator for 5 d. The culture medium of each flask was transferred to a 10-ml centrifuge tube, and some culture medium was added into the culture flask. After adherent cells were scraped with a cell scraper, cells were completely dispersed and transferred into a new culture flask. After attaching for 1 h, non-adherent cells and floating cells in supernatant were collected and centrifuged at 250×*g* for 10 min. Supernatant was then discarded, and cells were washed twice with culture medium. After re-suspension, cells were counted. Semi-solid cultures of granulocyte/monocyte colony-forming units (CFU-GM), erythroid burst-forming units (BFU-E), and megakaryocyte CFUs (CFU-Meg) were established according to the published literature [20].

### Transplantation of VCAM-1 gene–modified HUCBSCs in a hematopoietic damage model

HUCBSCs were injected through nude mouse tail veins. The mice were divided into four groups. The control group was infused with normal saline, the HUCBSC group with 1×10^5^ HUCBSCs, the VCAM-1HUCBSC1 group with 1×10^5^ VCAM-1–modified HUCBSCs, and the VCAM-1HUCBSC2 group with 1×10^6^ VCAM-1–modified HUCBSCs. After injection, these experimental nude mice were housed in SFP animal rooms in Xinqiao Hospital.

### Detection of VCAM-1 in nude mouse bone marrow stromal cells

#### Immunocytochemical detection of VCAM-1 in nude mouse bone marrow stromal cells

Bone marrow stromal cells from nude mice before transplantation and on days +1, +7, +14, and +21 were cultured on cover slips for 7 d. Cells were then washed twice with 0.01 mol/L PBS (pH 7.4), fixed in acetone for 5–10 min, and incubated in 100 ul of peroxidase-blocking solution for 10 min at 37°C. After washing with PBS three times for 3 min each, cells were incubated with 100 ul of non-immunized animal serum at 37°C for 10 min. Cells were washed three times with PBS (5 min per wash), PBS was discarded, and cells were incubated with rabbit anti-human VCAM-1 monoclonal antibody at 37°C for 60 min. After washing with PBS five times for 3 min each, cells were incubated with 100 ul of biotin-labeled secondary antibodies at 37°C for 10 min. Cells were washed with PBS three times for 3 min each and incubated with 100 ul of streptomycin avidin peroxidase at 37°C for 10 min. After three more washes with PBS for 3 min each, cells were incubated with 200 ul of DAB solution, then observed under a microscope for 3–10 min until color developed. After washing with tap water for 5 min, cells were stained with hematoxylin solution for 2 min and washed again with tap water, acid-alcohol solution rapidly, tap water, ammonia-water solution, and tap water. Cells were then dehydrated successively with 70% alcohol once, 95% alcohol once, and anhydrous alcohol three times for 1 min each. Finally, cells were cleared with xylene solution, mounted using Permount, and observed under a microscope.

### Flow cytometry of VCAM-1 in nude mouse bone marrow stromal cells [21]

After the culture medium was discarded, cells were washed with RPMI1640 twice and digested with 0.25% trypsin containing 0.02% ethylenediaminetetraacetic acid at 37°C for 10 min or until space between cells became widened and cells began to become round. After the trypsin was discarded, cells were dispersed by gently pipetting in an appropriate amount of RPMI and centrifuged at 1,000 rpm for 10 min. The supernatant was then discarded, and cells were re-suspended in 4°C PBS at 1×10^6^ cells/ml; the survival rate of cells was over 95%. The primary antibody (VCAM-1) was added into 1 ml of cell suspension at the final concentration of 20 g/ml and incubated at 4°C for 30 min. PBS was added in place of primary antibody in the negative control group. Cells were washed with buffer twice; after residual liquid was removed, the cell pellet was dispersed by gently tapping. Fluorescein isothiocyanate–labeled secondary goat anti-mouse IgG was then added and incubated at 4°C for 30 min. After two washes with buffer, cells were mixed with 1 ml of buffer and were detected using a flow cytometer.

### Observation of the effect of transplantation of VCAM-1–modified HUCBSCs on the reconstruction of the function of HIM

#### Changes in the hemogram in nude mice

Twenty microliters of tail vein blood from each transplantation group was collected on days +1, +3, +5, +7, +10, +14, and +21 after transplantation. Each blood sample was mixed with 0.2 ml of dilution buffer and was measured using a blood counter.

#### Changes in the myelogram

One nude mouse from each transplantation group on days +1, +7, +14, and +21 was sacrificed using neck dislocation for myelographic examination.

### Bone marrow stromal CFU-F detection [22]

One nude mouse from each group on days +7, +14, and +21 was sacrificed using neck dislocation. After soaking in 75% alcohol for 5 min, bilateral femoral and tibial bones were peeled off using rongeurs and smooth forceps and placed in IMDM containing 10% newborn bovine serum (NBS). Bone marrow was washed out using a 1-ml syringe with a 7-gauge needle; next, 7-, 5-, and 4-gauge needles were used sequentially to filter single cells from bone marrow, and 7-, 5-, and 4-gauge needles were used sequentially to filter cells into single-cell suspensions. Cells were then inoculated onto IMDM containing 10% NBS, 10% HS, 10^−6^ mol/L hydrocortisone, and 100 U/ml penicillin and streptomycin at 1×10^6^ cells/cm^2^. Cells were incubated at 37°C in a 5% CO_2_ incubator. After 48 h, the supernatant was discarded; half of the medium was changed every week, and each sample was cultured in five flasks.

### Semi-solid culture of bone marrow mononuclear cells, CFU-GM, BFU-E, and CFU-Meg from nude mice [23]

One nude mouse from each group on days +7, +14, and +21 was sacrificed using neck dislocation. Mononuclear cells from bilateral femoral bones were cultured *in vitro* for CFU-GM, BFU-E, and CFU-Meg. Each flask had 2×10^5^ mononuclear cells added to it.

### Dynamic observation of bone marrow pathological slices after HUCBSC transplantation

Preparation of bone marrow pathological slices:

One nude mouse from day +21 was sacrificed by neck dislocation, and bilateral tibial bones were removed to make bone marrow pathological slices. Samples were placed in Bouin fixative solution (picric acid fixative) for 30–60 min and dehydrated in 69%, 70%, 80%, 95%, and 100% (twice) alcohol. Samples were embedded in Hemapun865 plastic embedding medium. Hemapun865 solution A was first added, followed by an incubation in a refrigerator for 1–2 h. Then, several drops of solution B was added using a 1-ml syringe with an 8-gauge needle and mixed immediately with solutions A, followed by an incubation at room temperature for 20 min until the solutions had self-polymerized. After embedding, samples were cut into blocks, sectioned, stained with HGF, and examined under a microscope.

Preparation of bone marrow pathological slices suitable for immunofluorescence microscopy:

The high heat generated during preparation of general bone marrow pathological slices would denature proteins, damage tissues and cells, and destroy the activities of many enzymes and antigenicity. To observe whether HUCBSCs were really transplanted into living tissues in bone marrow, we used a new technique to prepare bone marrow pathological slices, the Hemapun948 cold vacuum embedding technique. The technique not only can achieve a slow and uniform polymerization but also can suppress the heat reaction induced by the process of rapid polymerization, thus preventing heat damage and preserving many cellular antigenic components. The results indicated that the cells observed under a light microscope were HUCBSCs homing back to the bone marrow through veins.

For embedding, bone marrow pathological tissues were fixed in pre-cooled 4°C Bouin fixative solution for 1 h, washed with 0.1 ml/L sodium dimethylarsinate buffer in a 4°C refrigerator for 30 min, and dehydrated at 4°C in a gradient of alcohol concentrations for 10 min in each concentration. Living tissue blocks were then placed on the bottom of a mold with 2 ml of solution A in a type 1354 vacuum dryer for 2–4 h in a 4°C refrigerator. Two to three drops of alcohol were then added into the mold with living tissue blocks at 4°C to make complete embedding solution; the polymerization was completed under vacuum conditions.

### Statistical analysis

Data were represented as mean values with standard deviation. Statistical significance was analyzed using the Student's t test. P values less than 0.05 were considered significant.

## Results

### HUCBSC culture

#### Collection of cord blood and separation and purification of CD34^+^ cells in cord blood

Twenty samples of cord blood were collected in the study. The average volume was 85±28.6 ml, the number of nucleated cells was 1.85±0.72×10^7^ cells/mL, the number of mononuclear cells purified using Ficoll was 2.43±1.37×10^8^, the number of CD34^+^ cells separated using magnetic-activated cell sorting was 5.47±2.29×10^6^, and the purity determined by flow cytometry was 0.91±0.04.

#### Dynamic observation of the growth of HUCBSCs

**Figure 1:**
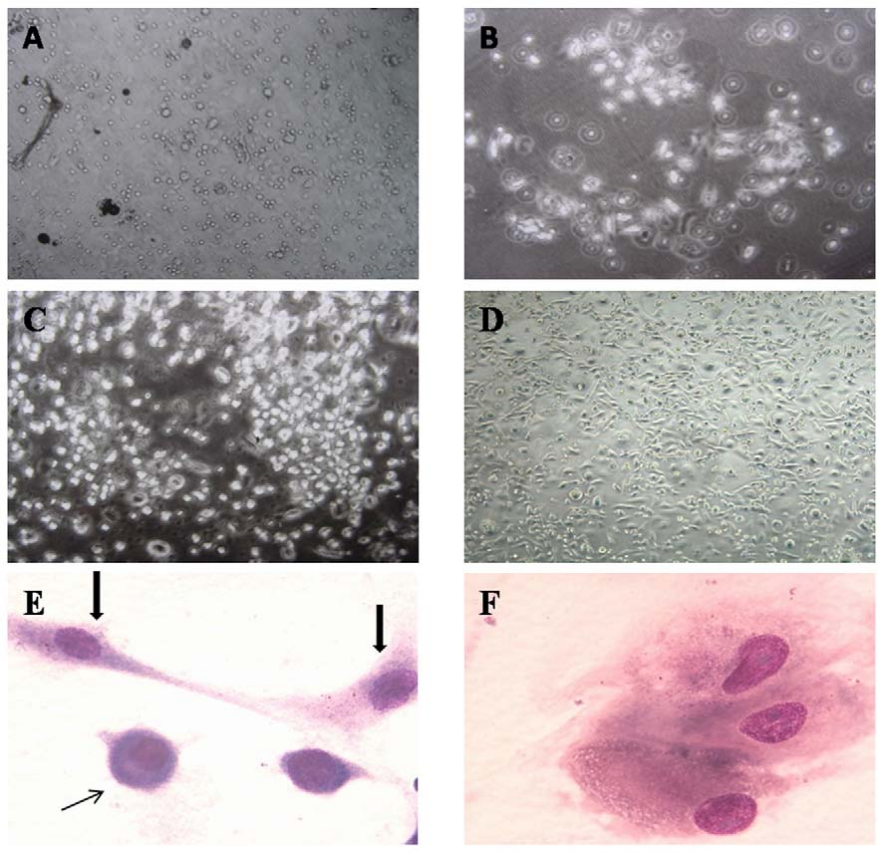
Dynamic observation of the growth of Dexter-cultured HUCBSCs. 1-A: Dexter-cultured HUCBSCs formed adherent cells on day 3. 1-B: Dexter-cultured HUCBSCs formed smaller stromal cell colonies on day 14. 1-C: There were larger and more stromal cell colonies in Dexter-cultured HUCBSCs on day 21. 1-D: Stromal cells became confluent in Dexter-cultured HUCBSCs on day 28. (inverted microscope, ×100). 1-E: Fibroblast-like (thick arrow) and “small-round” cells (thin), (Wright's staining, inverted microscope×1000). 1-F: Macrophage-like cells, (inverted microscope ×1000).

After 3–4 d of culture in the Dexter culture system, adherent cells could be observed among the CD34^+^ cells from cord blood and were round, triangular, or irregularly shaped (Fig. 1-A). After 9–14 d (average 11.2 d), stromal cell colonies began to appear (Fig. 1-B). The number of colonies reached its maximum after 15–22 d (average 19.6 d) (Fig. 1-C). With increasing culture time, cell volume gradually increased and cells gained different morphologies such as round, oval, or spindle shapes. Cell types were mainly fibroblast-like or macrophage-like. After 28 d of culture, adherent cells became confluent (Fig. 1-D). The major presentations of morphology features of HUCBSCs were as follows: a. Fibroblast-like cells, which accounted for 56.5%; the cells were spindle-shaped with abundant cytoplasm that was purple in color, thicker chromatin, and a non-obvious nucleolus (Fig. 1-E, thick arrow). b. Macrophage-like cells, which accounted for 38%; the cells were round and bigger, processes on cell membrane were chimeric, the cytoplasm was abundant, the chromatin was thick, and the nucleolus was obvious (Fig. 1-F). c. “Small-round” cells, which accounted for 5.5%; the cells were smaller and looked like lymphocytes, the cytoplasm was blue, the chromatin was thin, and the nucleolus was obvious (Fig. 1-E, thin arrow).

#### Transfection of the Ad-VCAM-1-EGFP adenovirus vector into HUCBSCs

**Figure 2:**
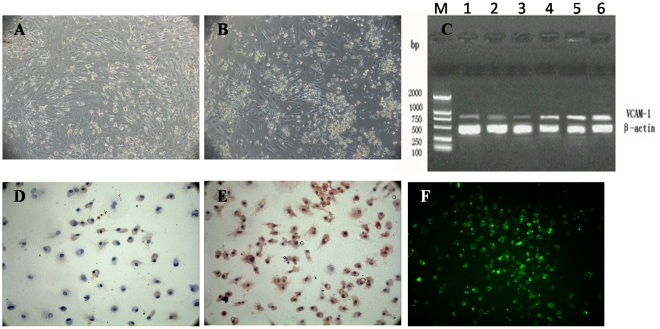
HUCBSCs transfected with the Ad-VCAM-1-EGFP adenovirus plasmid. 2-A: HUCBSCs before VCAM-1 transfection. 2-B: HUCBSCs after VCAM-1 transfection. 2-C: Detection of VCAM-1 expression before and after transfection using RT-PCR, M: DNA marker; 1,2,3: before transfection; 3,4,5: after transfection. 2-D: The weak expression of VACM-1 in HUCBSCs before transfection. 2-E: The increased expression of VCAM-1 in HUCBSCs after transfection. 2-F: Immunofluorescence detection of VCAM-1 of HUCBSCs after transfection.

The percentage of live HUCBSCs after VCAM-1 transfection was 94%; the percentage of live cells in the control cells not transfected with VCAM-1 was 97%. The stained HUCBSCs before and after transfection are shown in Fig. 2-A and 2-B. Semi-quantitative RT-PCR showed that the expression of VCAM-1 mRNA significantly increased after transfection (Fig. 2-C). Immunocytochemistry showed that the positive rate of VCAM-1 protein expression significantly increased after transfection (Fig. 2-D, E). The percentage of green fluorescent cells was counted under a fluorescence microscope (Fig. 2-F). The efficiency of liposome-mediated transfection of recombinant plasmids in this study was 40–70%, with an average of 54.6%.

#### The effect of HUCBSCs on the in vitro proliferation of cord blood CD34^+^ cells

**Figure 3:**
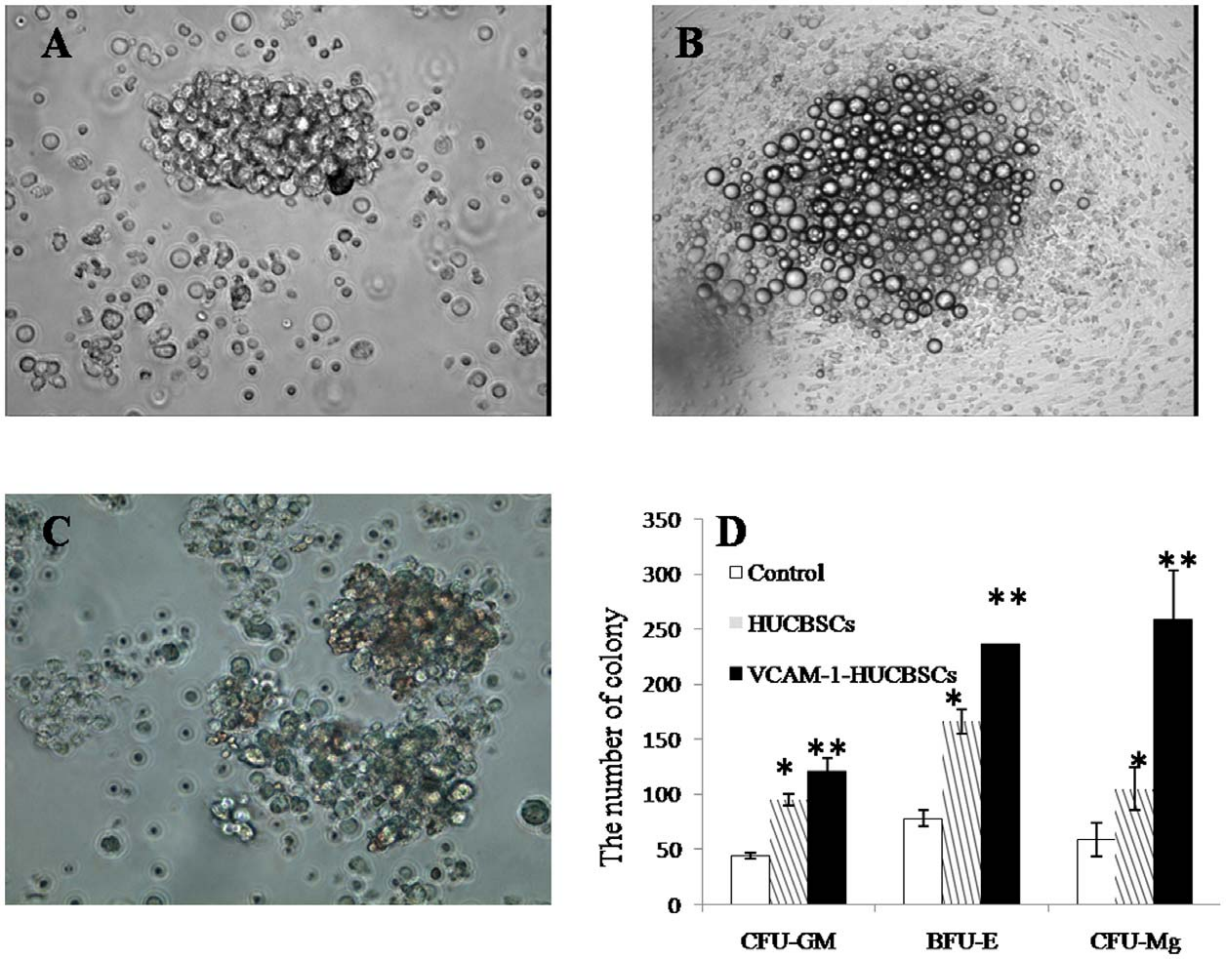
The effect of HUCBSCs on the *in vitro* proliferation of cord blood CD34^+^ cells. 3-A: Culture of CFU-GM from human cord blood CD34^+^ cells. 3-B: Culture of BFU-E from human cord blood CD34^+^ cells. 3-C: Culture of CFU-Meg from human cord blood CD34^+^ cells. 3-D: Comparison of numbers of colonies in the three groups. * compared with control group, P<0.01; ** compared with control and HUCBSCs groups, p<0.01.

On days 1–3, myeloid/mononuclear, erythroid, and megakaryocytic cell lineages grew as single cells; they began to proliferate after day 4. At first, there were cell clusters of 3–5 cells; colonies could be observed after day 7, and colonies significantly increased during days 10–12. CFU-GM were colorless cell masses with more than 50 cells; cells were tightly connected and showed dense colonies (Fig. 3-A). BFU-E were erythroid progenitor cell clusters with more than 50 cells that were hemoglobinized; cells in each colony were round with small volume and were tightly connected, and because hemoglobin was synthesized in the cytoplasm, colonies were dark red (Fig. 3-B). CFU-Meg were cell masses with more than 20 cells; these cells were larger in size, with irregular shapes (Fig. 3-C). After 14 days, colonies gradually decreased in size, and cells degenerated. The number of colonies of each lineage in groups supported by HUCBSCs was higher than that in each control group without HUCBSC feeder layers (p<0.01), while the number of colonies of each lineage in the VCAM-1–modified HUCBSC group was higher than that in the HUCBSC-supported group and control group without a HUCBSC feeder layer (p<0.01) (Fig. 3-D).

#### Establishment of a nude mouse model for HIM damage

Dynamic changes of the nude mouse hemogram after 3.5 Gy, 5 Gy, 6.5 Gy, and8 Gy irradiation

**Figure 4:**
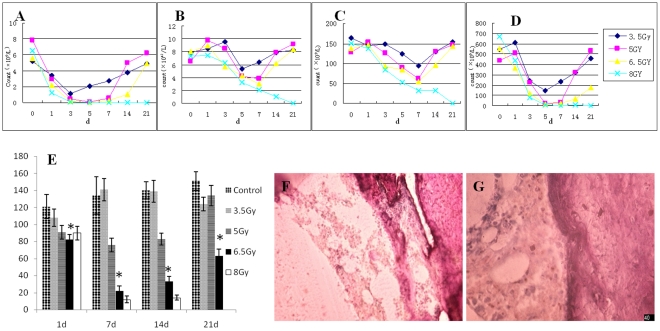
Dynamic observation of the nude mouse hemogram, bone marrow stromal CFU-F count, and bone marrow pathological slices after different doses of irradiation. 4-A: WBC counts. 4-B: RBC counts. 4-C: Hemoglobin counts. 4-D: PLT counts. 4-E: CFU-F counts in bone marrow stromal cells, * compared with control, 3.5 and 5 Gy groups, P<0.01. 4-F: Nude mouse bone marrow pathological slices after 6.5 Gy irradiation. 4-G: Nude mouse bone marrow pathological slices after 8 Gy irradiation.

The changes in the hemogram differed among the nude mice that received the four different doses of irradiation. In the 3.5 Gy group, the white blood cell (WBC) and platelet (PLT) counts were the lowest on day +3, and the red blood cell (RBC) count was the lowest on day +5; after that, indicators of hemogram rapidly recovered. The WBC and PLT counts of the 3.5 Gy, 5 Gy, and 6.5 Gy groups were the lowest on day +5, and the RBC counts of the 5 Gy and 6.5 Gy groups were the lowest on day +7. The recovery in the 5 Gy group was faster than that in the 6.5 Gy group. One out of three nude mice died in the 6.5 Gy group. When the radiation dose increased to 8 Gy, the hematopoietic function of nude mice could not be recovered, which led to the death of nude mice, and experiments could not be continued (Fig. 4-A–E).

#### Dynamic observation of bone marrow pathological slices from nude mice after 3.5 Gy, 5 Gy, 6.5 Gy, and 8 Gy irradiation

We focused on trabecular bone tissues because the morphology of stromal cells and hematopoietic precursor cells and their locations could be observed in these tissues. With increased radiation dose, characteristic changes in the bone marrow pathological slices, especially in tissues around trabecular bone tissues, were apparent: cell bodies of bone marrow stromal cells close to trabecular bone became larger, with loose and disordered arrangement; the number of scattered hematopoietic progenitor cells decreased; and, the number of cells with ring-shaped nuclei increased in bone marrow. These phenomena were most obvious in the 6.5 Gy and 8 Gy groups (Fig. 4-F and G).

Overall, the results after the four different doses of irradiation showed that after 8 Gy irradiation, nude mice died because hematopoietic function could not be recovered in time, leading to the termination of experiments. The hematopoietic function of nude mice in the 3.5 Gy, 5 Gy, and 6.5 Gy groups basically could be recovered, although longer time was needed; most mice housed in SPF animal rooms survived independently. Bone marrow stromal CFU-F detection showed that the function of bone marrow stromal cells from nude mice in the 5 Gy and 6.5 Gy groups had significant damage; therefore, irradiated animals could be used as a model for HIM damage. To better observe the effects of HUCBSC transplantation on the improvement of HIM function and repair of hematopoietic damage while excluding the effect of HSC infusion on the experiments, we chose 6.5 Gy in the subsequent studies for the radiation dose in the nude mouse model for HIM damage. Furthermore, *in vitro*–expanded HUCBSCs were infused to observe their effect on the repair of HIM function in nude mice and the recovery of hematopoietic damage.

#### The expression of VCAM-1 in nude mouse stromal cells at different time points after HUCBSC transplantation

**Figure 5:**
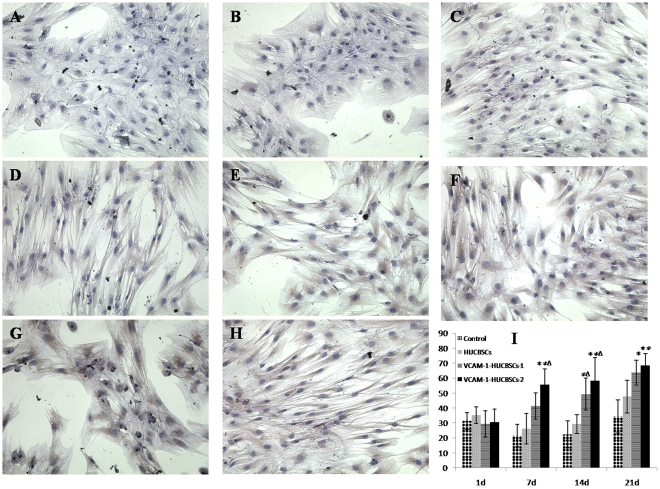
Expression of bone marrow stromal cell VCAM-1 in nude mice after HUCBSCs transplantation. 5-A: control group +14 d; 5-B: control group +21 d; 5-C: HUCBSCs group +14 d; 5-D: HUCBSCs group +21 d; 5-E: VCAM-1-HUCBSCs-1 group +14 d; 5-F: VCAM-1-HUCBSCs-1 group +21 d; 5-G: VCAM-1-HUCBSCs-2 group +14 d; 5-H: VCAM-1-HUCBSCs-2 group +21 d (immunohistochemistry under inverted microscope×400); 5-I: flow cytometry for the expression of VCAM-1, * compared with control group, P<0.05, * *compared with control group, P<0.01,^Δ^ compared with HUCBSCs group P<0.05.

Immunocytochemistry showed that the expression of VCAM-1 in *in vitro*–cultured nude mouse stromal cells on days +14, and +21 after transplantation decreased in the control and HUCBSC transplantation groups; the expression still had not recovered on day +21 (Fig. 5-A, B, C, D). The expression of VCAM-1 in nude mouse bone marrow stromal cells in the VCAM-1-HUCBSC-1 and VCAM-1-HUCBSC-2 groups on day +14 was significantly higher than that in the control and HUCBSC transplantation groups (Fig. 5-E and F). With an increased number of transplanted cells and transplantation time, the expression of VCAM-1 showed an increasing trend, with the expression of VCAM-1 highest in the VCAM-1-HUCBSC-2 group (Fig. 5-G and H). Flow cytometric results also confirmed the above trends (Fig. 5-I).

#### Effects of VCAM-1 gene–modified HUCBSCs on the repair of hematopoietic function

**Figure 6:**
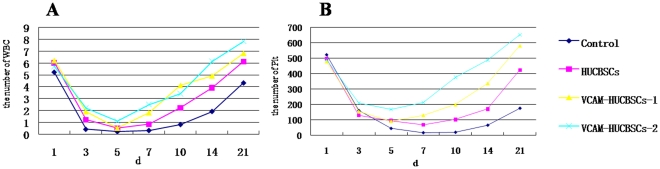
Dynamic changes in peripheral WBC and PLT counts in nude mice after transplantation. 6-A: Dynamic changes in WBC counts after transplantation. 6-B: Dynamic changes in PLT counts after transplantation.

Changes in peripheral WBC and PLT counts in nude mice. The peripheral WBC count of nude mice in each group rapidly decreased 1 d after transplantation, reached the lowest level on day +5, and then gradually increased. On day +21, the WBC levels in the HUCBSC group and VCAM-1-HUCBSC-1 group recovered to the day +1 level. On day +14, the WBC level of nude mice in the VCAM-1-HUCBSC-2 group returned to the day +1 level. On day +21, the WBC level was significantly higher than that in the other three groups. On day +21, the WBC count in the control group had not returned to the day +1 level (Fig. 6-A).

The PLT counts in each group rapidly decreased on day +1 after transplantation. The PLT counts of the control group and HUCBSC group reached the lowest levels on day +7 and then gradually increased; on day +21, the PLT levels still had not returned to the day +1 level. The PLT counts of the VCAM-1-HUCBSC-1 and VCAM-1-HUCBSC-2 groups reached the lowest levels on day +5, while the PLT counts on day +21 were higher than that on day +1. The VCAM-1HUCBSC2 group had the highest recovery level (Fig. 6-B).

#### Dynamic changes in the myelogram of nude mice after HUCBSC transplantation using 6.5 Gy irradiation

**Figure 7:**
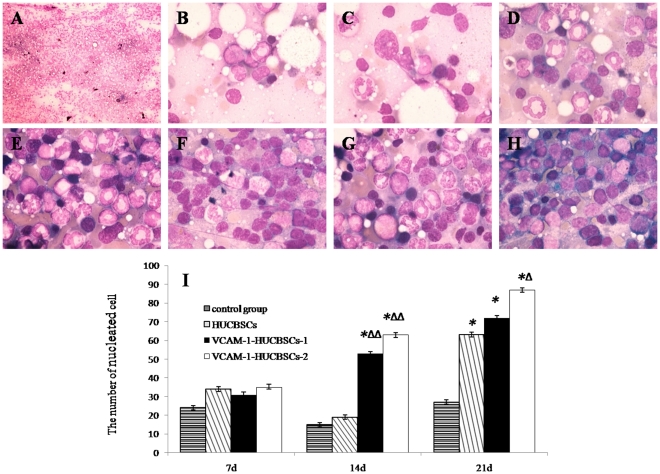
Dynamic changes in the myelogram in nude mice after HUCBSCs transplantation after 6.5 Gy irradiation. 7-A: The degree of bone marrow hyperplasia in control nude mice on day +1 after transplantation. 7-B: Myelogram of control nude mice on day +14. 7-C: Myelogram of control nude mice on day +21. 7-D: Myelogram of nude mice in the HUCBSC group on day +14. 7-E: Myelogram of nude mice in the HUCBSC group on day +21. 7-F: Myelogram of nude mice in the VCAM-1-HUCBSC-1 group on day +14. 7-G: Myelogram of nude mice in the VCAM-1-HUCBSC-2 group on day +7. 7-H: Myelogram of nude mice in the VCAM-1-HUCBSC-1 group on day +21. 7-I: Counting of bone marrow nucleated cells in nude mice after HUCBSCs transplantation, * compared with control group, P<0.01; ^Δ^ compared with HUCBSCs group P<0.05; ^ΔΔ^ compared with HUCBSCs group, P<0.01.

Bone marrow proliferated more actively in mouse than in healthy human. Because the number of cells is excessively large under the low-powered microscope and therefore is difficult to count, we compared the difference of bone marrow hyperplasia among four groups by counting nucleated cells in each oil immersion lens field. Bone marrow hyperplasia in control nude mice on day +1 after irradiation was basically normal (Fig. 7-A). Bone marrow hyperplasia significantly decreased on day +7 and day +14, and the number of nucleated cells was lowest in day +14(Fig. 7-B). The level of hyperplasia had not fully recovered to normal on day +21 (Fig. 7-C). Bone marrow hyperplasia in the HUCBSC transplantation group started to decrease on day +7, reached its lowest level on day +14 (Fig. 7-D), and recovered to the normal level on day +21 (Fig. 7-E). The bone marrow hyperplasia in the VCAM-1HUCBSC1 group was already recovered on day +14 (Fig. 7-F), while the bone marrow hyperplasia in the VCAM-1-HUCBSC-2 group only had slightly decreased on day +7 (Fig. 7-G) and recovered to the normal level by day +14 (Fig. 7-H). Bone marrow morphological visualization of the control group and VCAM-1-HUCBSC-2 group on day +14 and day +21 showed a significant difference in the numbers of nucleated cells in bone marrow in these two groups. The bone marrow hyperplasia in the HUCBSC transplantation group was also significantly higher than that in the control group on day +21 (Fig. 7-I).

6.3 The effects of nude mouse bone marrow stromal cells on CFU-F, myeloid, erythroid, and megakaryocytic lineage cells after transplantation of HUCBSCs

**Figure 8:**
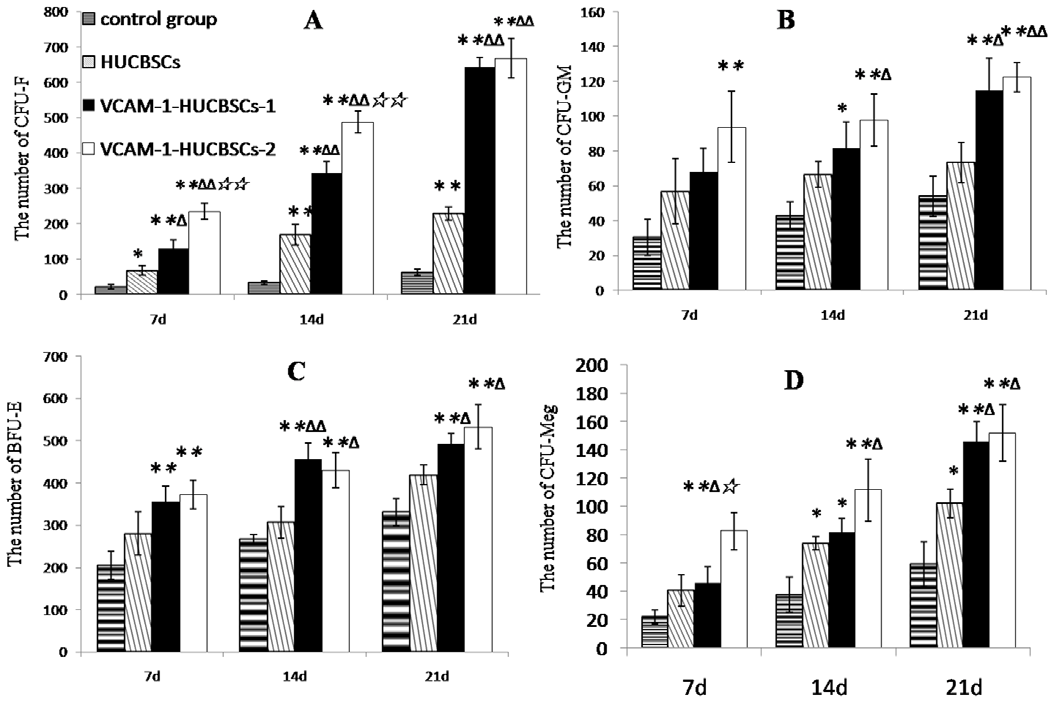
Effects of nude mouse bone marrow stromal cells on CFU-F, myeloid, erythroid, and megakaryocytic lineage cells after transplantation of HUCBSCs. 8-A: changes of CFU-F in nude mice at different time points after HUCBSCs transplantation. 8-B: changes of CFU-GM in nude mice at different time points after HUCBSCs transplantation. 8-C: changes of BFU-E in nude mice at different time points after HUCBSCs transplantation. 8-D: changes of CFU-Meg in nude mice at different time points after HUCBSCs transplantation.. * compared with control group, P<0.05;** compared with control group, P<0.01; ^Δ^ compared with HUCBSCs group P<0.05; ^ΔΔ^ compared with HUCBSCs group, P<0.01; ^⋆^ ccompared with VCAM-1-HUCBSCs-1 group P<0.05; ^⋆⋆^compared with VCAM-1-HUCBSCs-1 group P<0.01.

Figure 8 shows that after HUCBSC transplantation, the numbers of CFU-F, CFU-GM, BFU-E and CFU-Meg were the lowest on day +7 after transplantation and then gradually increased. At any time point, the numbers in the transplantation group were always higher than those in the control group; the numbers in the VCAM-1-HUCBSC-2 group were significantly higher than those in the HUCBSC group and VCAM-1-HUCBSC-1 group (Fig. 8).

#### Dynamic observation of bone marrow pathological slices after HUCBSC transplantation

**Figure 9:**
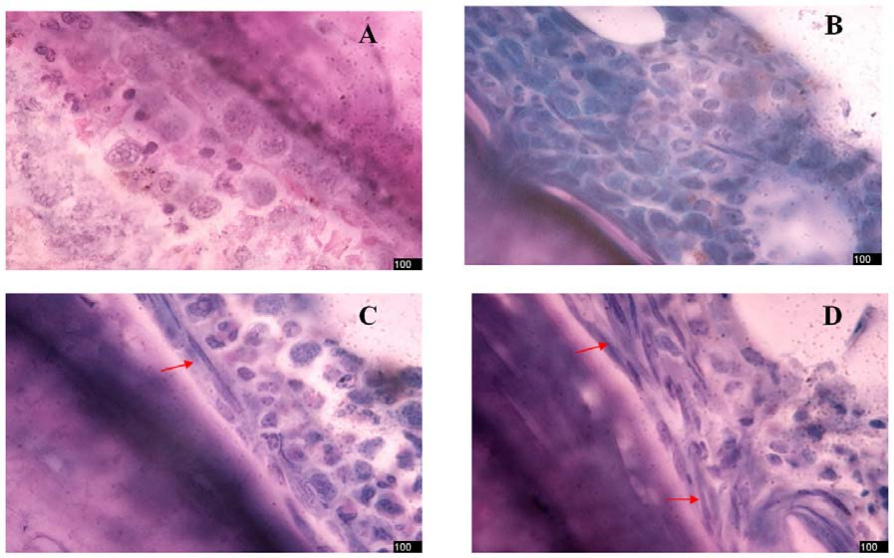
Observation of the pathological slides of nude mouse bone marrow under light microscope on day +21 after transplantation. 9-A: control group; 9-B: HUCBSCs group; 9-C: VCAM-1-HUCBSCs-1 group; 9-D: VCAM-1-HUCBSCs-2 group, (HGF staining, inverted microscope, ×100). Red arrows represent new fibroblast-like cells.

Observation using a light microscope. Compared to the control group on day +21, adherent bone marrow stromal cells that were neatly arranged in parallel could be observed around trabecular bone tissues in bone marrow pathological slices from the HUCBSC and VCAM-1-HUCBSC groups. There were large amounts of nucleated cells close to stromal cells around trabecular bone, which were mainly proliferating progenitor cells, indicating that the hematopoietic function of bone marrow was very strong. This phenomenon was most prominent in the VCAM-1-HUCBSC groups and was most significant in the group with more infused cells (Fig. 9).

#### Immunofluorescence microscopy observations

**Figure 10:**
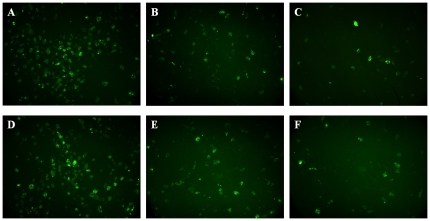
Observation of the fluorescences in the bone marrow pathological slices of the VCAM-1HUCBSC groups. 10-A: VCAM-1-HUCBSC-1 group on day +7 after transplantation; 10-B: VCAM-1-HUCBSC-1 group on day +14 after transplantation; 10-C: VCAM-1-HUCBSC-1 group on day +21 after transplantation; 10-D: VCAM-1-HUCBSC-2 group on day +7 after transplantation; 10-E: VCAM-1-HUCBSC-2 group on day +14 after transplantation; 10-F: VCAM-1-HUCBSC-2 group on day +21 after transplantation (fluorescence microscope ×100).

Fluorescence was not observed in pathological slices from the control group or HUCBSC group at any time point. Green fluorescence was observed in the bone marrow pathological slices of the VCAM-1HUCBSC groups on days +7, +14, and +21 after transplantation. With increasing time, the intensity and density of fluorescence gradually decreased, reaching their minimum levels on day +21 after transplantation. At the same time point, the intensity and density of immunofluorescence in the VCAM-1-HUCBSC-2 group were significantly higher than those in the VCAM-1-HUBCSC-1 group (Fig. 10).

## Discussion

In our previous study, we had successfully cultured and isolated HUCBSCs and demonstrated that they could secret hemopoietic growth factors such as GM-CSF, TPO, and SCF, which provided a new hematopoietic resource [12]. In the current experiment, we initially established a co-culture system for HUCBSCs and umbilical cord blood CD34^+^ cells. The results of co-culture of HUCBSCs and cord blood CD34^+^ cells in this study showed that when using HUCBSCs as the adherent layer, the formation of colonies of each hematopoietic lineage significantly increased; erythroid, myeloid, and megakaryocytic lineages all formed more colonies than those in the control groups without HUCBSCs as feeder layers 12 d after co-culture. One possible cause of these results is that HUCBSCs secrete cytokines that directly contact the hematopoietic cells and regulate their growth. However, these results confirm that HUCBSCs can support the formation of the HIM. The exact mechanism of the interaction between the HIM formed by stromal cells and hematopoietic parenchymal cells is still unclear; the current consensus is that it is due to the direct contact between stromal cells in the HIM and HSCs or the important function of secreted cytokines in the regulation of hematopoiesis. However, the specific forms and the mechanisms of the resultant signal transduction pathways have not been elucidated. Yanai [24] found that when HSCs and bone marrow stromal cells were co-cultured, HSCs had a tendency of migrating toward stromal cells, while more mature hematopoietic cells would only move to the surface or float in culture medium. Similar results have also been observed in studies using cell lines. When the HSC line THS119 was inoculated onto stromal cell layers, THS119 cells attached to the surface of stromal cells and migrated to the cobblestone-like colony-forming region of the stromal cell layers before proliferation. Wagner also found that hematopoietic cells formed large amounts of processes that connected to stromal cells at multiple points, and some processes reached the inner layer of stromal cells. When stromal cells took up dyes, the dyes entered the adjacent hematopoietic cells and stromal cells, indicating that the direct contact between stromal cells and hematopoietic cells had the function of transmitting and exchanging signaling molecules. The contact was achieved through the adhesion molecules on the surface of stromal cells and hematopoietic cells.

CAMs are the molecular bases of homing and information transfer in HSCs/HPCs [25]. The adhesion of stem cells and stromal cells is important for hematopoietic growth and development. For the functions of hematopoietic stromal cells, the expression of important CAMs, such as CD106 (VCAM-1), CD29 (integrin), and CD44, is helpful for stromal cells to exert their hematopoietic regulation functions [26], [27], [28]. CAMs can promote the homing of stromal cells after transplantation and play important roles in lymphocyte aggregation and immune response regulation. Fibronectin (Fn), laminin (Ln), and type IV collagen are not only components of intercellular substances but are also important components of the ECM produced by stromal cells such as fibroblasts, endothelial cells, and macrophages. More importantly, they provide material bases for transmitting information between cells [11]. Our previous studies confirmed that *in vitro*–cultured HUCBSCs express CAMs such as CD29 and CD44 and secrete ECM components such as Fn, Ln, and type IV collagen, suggesting that HUCBSCs have the material bases of hematopoietic functional regulation. The expression of VCAM-1 in these cells was weaker, and VCAM-1 plays important roles in regulating hematopoiesis in stromal cells and is upstream of CAM activity (such as CD29) [12], therefore, Ad-VCAM-1-EGFP adenovirus plasmid–transfected HUCBSCs were established in this study to observe the hematopoietic supporting functions of HUCBSCs with increased VCAM-1 expression. Compared with other cultures, VCAM-1–modified HUCBSC adherent cell layers significantly increased the number of colonies formed by CD34^+^ cells; the numbers of erythroid, myeloid, and megakaryocytic progenitor cell colonies formed after 12 d of co-culture were all significantly higher than in the group with HUCBSC adherent cells layers and in the control group without HUCBSC adherent layers. These results indicate that increased expression of VCAM-1 in HUCBSCs can increase the specific interaction between VCAM-1 and its receptor α4β1 integrin, increase the adhesion of HSCs/HPCs, and localize HSCs/HPCs in the HIM. Therefore, HSCs/HPCs and stromal cells develop a closer association, which is conducive to the regulation of HSC/HPC proliferation and differentiation by signal transduction and secretion of hematopoietic growth factors and ECM components, thus leading to improved hematopoietic supporting function.

To observe the effect of HUCBSC transplantation on the recovery of HIM function, which promotes the repair of hematopoietic damage, we explored different doses of radiation on the damage of mouse HIM to establish an animal model using an appropriate dose of radiation to damage the HIM. Hematopoietic tissues are sensitive to radiotherapy; studies on hematopoietic damage usually use ^60^Co γ-irradiated mice to establish an animal model for damaged hematopoietic function. However, few studies have reported on the appropriate radiation dose for damaging the HIM. We first set up four radiation groups, 3.5, 5, 6.5, and 8 Gy, and observed the changes in nude mouse hemogram and the formation of bone marrow CFU-F at different time points after irradiation. The expression of VCAM-1 in nude mouse bone marrow stromal cells after irradiation was also detected using flow cytometry. Changes in hemogram were different after different doses of irradiation. The WBC and PLT counts of nude mice in the 3.5 Gy group were the lowest on day +3, and RBC count was the lowest on day +5; the indictors of the hemogram were then rapidly recovered. The WBC and PLT counts of nude mice in the 3.5, 5, and 6.5 Gy groups were the lowest on day +5, and RBC counts in the 5 and 6.5 Gy groups were the lowest on day +7. The recovery rate of the 5 Gy group was faster than that of the 6.5 Gy group, and one out of three nude mice died in the 6.5 Gy group. When the dose increased to 8.0 Gy, the hematopoietic function of nude mice had not recovered even on day +21; all mice died, and experiments could not be continued.

When the radiation dose was 8 Gy and there was no support from HSC transplantation, the nude mice died because hematopoietic dysfunction could not be repaired, so experiments could not be continued. The hematopoietic function of nude mice in the 3.5 Gy, 5 Gy, and 6.5 Gy groups could be self-recovered within 21 d after irradiation, and most nude mice housed in SFP animal rooms could survive independently; only one nude mouse died, and experiments could be continued. The bone marrow stromal CFU-F and VCAM-1 measurement showed that the changes of CFU-F and VCAM-1 in the 3.5 Gy group were not significant. The nude mouse bone marrow stromal CFU-F and VCAM-1 were significantly damaged in the 5 Gy and 6.5 Gy groups compared to those in the control group. The degree of HIM damage in the 6.5 Gy group was more severe after irradiation. The number of CFU-F and the expression of VCAM-1 were significantly different from those in the other two groups; therefore, the irradiation of nude mice using 6.5 Gy could achieve the purpose of causing HIM damage and would not cause animal death, allowing the experiments to continue.

The subsequent *in vivo* experimental results showed that infusion of either HUCBSCs alone or VCAM-1–modified HUCBSCs had significant effects on promoting the recovery of nude mouse WBC and PLT. The abilities to promote the recovery of hemogram and bone marrow hematopoietic function, ranging in order from strongest to weakest, were VCAM-1-HUCBSC-2, VCAM-1HUCBSC-1, and HUCBSC group. The control group had a longer myelogram recovery time, while the recovery time in the other three groups gradually shortened. The degrees of bone marrow hyperplasia in the HUCBSC, VCAM-1-HUCBSC-1, and VCAM-1-HUCBSC-2 groups were the lowest on day +21, day +14, and day +7, respectively. The CFU-GM, BFU-E, and CFU-Meg formation abilities in these three groups were stronger than that in the control group. In particular, when comparing the VCAM-1-HUCBSC-2 group to the control and HUCBSC groups, the numbers of nucleated cells, CFU-F, and nude mouse bone marrow HSCs/HPCs (CFU-GM, BFU-E, and CFU-Meg) on days 14 and 21 after HUCBSC transplantation were significantly different, indicating that VCAM-1 modification can increase the hematopoietic supporting functions of HUCBSCs.

Based on the current study, we believe that the effect of VCAM-1-modified HUCBSC in reconstructing HIM may be achieved via the following mechanisms: a) To directly repair the damaged HIM. The radiation-induced bone-marrow damage can directly cause the massive destruction of HIM. On day +21, as clearly shown in the bone marrow pathological slices, a large number of adherent bone marrow stromal cells were observed around trabecular bone tissues and the hematopoitic precursor cells massively proliferated near the stromal cells in the VCAM-1-HUCBSC groups; on the contrary, only a small number of stromal cells existed diffusely near the trabecular bone in the control group. Obviously, HUCBSC transplantation is helpful to repair the damaged HIM; b) To stimulate hematopoiesis by secreting hematopoietic growth factors. Our previous study [12] has found that HUCBSCs can express the mRNAs of hematopoietic growth factors including GM-CSF, TPO, and SCF. Compared with the bone marrow stromal cells, HUCBSCs had significantly lower mRNA expressions of SCF and GM-CSF and higher expression of TPO, demonstrating that HUCBSCs, similar to bone marrow stromal cells, are able to secrete and express hemopoietic growth factors and regulate the hematopoiesis process; and c) the hematopoiesis-supporting effect mediated by adhesion molecules. Research has found that the expression of VCAM-1 is relatively low in HUCBSCs, while VCAM-1 is an important adhesion molecule for the stromal cells to be involved in hematopoiesis-regulating. Obviously, this issue may directly affect the normal functions of HUCBSCs. Therefore, in our study, a VCAM-1 high-expressing vector was constructed and successfully transfected into HUCBSCs, and the results indicated that VCAM-1–modified HUCBSCs had stronger function in repairing HIM and promoting the recovery of hematopoietic damage.

Overall, these results suggest that VCAM-1–modified HUCBSCs have a stronger ability to repair HIM function and promote recovery of hematopoietic functions. These results demonstrate that VCAM-1 plays an important role in supporting hematopoiesis in the HIM and that our improvement on the new type of hematopoietic stromal cells was successful. These results may help the homing of HUCBSCs to bone marrow after transplantation and may help HUCBSCs to exert their hematopoietic-regulating functions. The above findings are helpful for investigating the effects of repairing HIM function on the rapid recovery of hematopoietic damage and its underlying mechanisms, which may provide a new route for finding more effective ancillary methods for treating hematopoietic damage.
